# The Antibacterial Activity of Yeasts from Unique Biocenoses

**DOI:** 10.32607/actanaturae.27527

**Published:** 2024

**Authors:** O. V. Shulenina, E. A. Sukhanova, B. F. Yarovoy, E. A. Tolstyko, A. L. Konevega, A. Paleskava

**Affiliations:** Petersburg Nuclear Physics Institute named by B.P. Konstantinov of National Research Centre “Kurchatov Institute”, Gatchina, 188300 Russian Federation; Peter the Great St. Petersburg Polytechnic University, St. Petersburg, 195251 Russian Federation; National Research Centre “Kurchatov Institute”, Moscow, 123182 Russian Federation

**Keywords:** antibiotics, biodiversity, yeast, collection, fluorescent reporter system

## Abstract

The replenishment of our stock of substances that possess a therapeutic potential is an important objective in modern biomedicine. Despite the important advances achieved in chemical synthesis, the natural diversity of organisms and microorganisms remains an important source of biologically active compounds. Here, we report the results of our study of a unique collection containing more than 3,000 samples of yeasts found on the Kamchatka Peninsula, the Kuril Islands, and Sakhalin Island, Russia. Since yeast and bacteria coexist in a variety of habitats and can interact with each other, we analyzed the antibacterial activity of the collection of yeast strains towards *E. coli *cells using a fluorescent bacterial reporter. It was uncovered that the Sakhalin strains for the most part stimulate bacterial growth, while most of the strains found on the Kamchatka Peninsula possess inhibitory properties. Moreover, the samples with the most pronounced antibacterial activity, identified as members of the genus *Cryptococcus (Naganishia)*, were found in a gorge in the vicinity of Pauzhetka village on the Kamchatka Peninsula on wormwood (*Artemisia vulgaris*) and thistle (*Onopordum acanthium*). Our data indicate that the combination of a plant and its growth site is important for the emergence of yeast strains capable of secreting antibacterial compounds.

## INTRODUCTION

The lack of strict oversight of the use of antimicrobial agents in medicine and livestock results in the emergence and spread of antibiotic-resistant pathogenic bacteria [1], making it necessary to look for new pharmaceuticals. The flora and fauna are an important source of biologically active compounds that have stood the test of time [2, 3]. Particular yeast strains have been studied well as they are heavily used in the food industry or as model organisms in research [4]. Some yeast metabolites can reduce the blood cholesterol level and act as immunomodulators or antifungal drugs [5]. The genera *Candida, Pichia, *and* Saccharomyces *exhibit antibacterial activity [5, 6, 7, 8]. However, yeasts are, biologically and chemically, incredibly diverse and yeast populations from isolated regions remain underinvestigated: they may possess unique properties [3].

In this paper, we study a unique collection of yeast gathered in the Russian Far East. The uniqueness of the collection has to do with the geography of these regions. Kamchatka and the Kuril Islands are parts of the Pacific Ring of Fire, which is characterized by heightened volcanic and seismic activity. The population of these regions is not numerous, and tourism is poorly developed; so, its nature is faintly exposed to the direct influence of civilization. The Kamchatka Peninsula, which is almost cut off from the mainland, has the highest concentration of volcanoes on Earth: 30 active ones out of more than 300 volcanoes. On the Kamchatka Peninsula and Kuril Islands, there is a dense network of mountain rivers rich in fresh water, lakes with high mineralization in volcanic areas heated by volcanic gases, and low-salted lagoon lakes. On the sides and in the craters of dormant volcanoes, there are hydrothermal, gas, and mud ejections of various temperatures and varying acidity, containing various natural inorganic compounds at high concentrations. Yeasts from Sakhalin exist at the same latitudes as species from the Kamchatka Peninsula and the Kuril Islands, but they are not affected by extreme environmental factors. Furthermore, unlike other species in the collection, the strains from Sakhalin were found closer to areas of human activity.

The objective of this study was to reveal and investigate isolates of the yeast collection that contain antimicrobial compounds in their culture liquids. We applied a reporter system that, apart from antimicrobial activity detection, allows one to sort potential antibacterials based on their mechanism of action. This double-reporter approach in identifying substances that cause ribosome stalling or induce the SOS response due to DNA damage was successfully used to screen a library of synthetized organic compounds [9] and actinomycetes extracts [10, 11, 12, 13]. The approach was designed for application on agar plates thereby minimizing the need for pipetting, reagents, and consumables. At the same time, fluorescent protein reporter assay on petri plates does not allow for a quantitative assessment of antibacterial action by exploiting mechanisms other than ribosome stalling or DNA damage. Additionally, the reporter sensitivity determined using a few antibiotics in a liquid medium was up to two orders of magnitude higher than that on agar [9].

Hence, in order to increase the chance of detecting even minor biologically active compounds in yeast culture liquids that could affect bacterial viability, we adapted a fluorescent double-reporter system to a liquid medium. To increase the sensitivity even further, we utilized an *E. coli *JW5503 bacterial strain lacking the *tolC *gene and coding for an essential component of several efflux systems [14]. The applied version of the reporter system was validated using 15 antibiotics with a known mechanism of action.

The assay was employed on 810 samples from the yeast collection, wherein 251 samples were from the Sakhalin collection and 559 samples were from the Kamchatka collection. The Sakhalin strains for the most part stimulated bacterial growth, while most of the strains from Kamchatka exhibited inhibitory properties. Our data point to the importance of the combination of the plant and its place of origin for the emergence of yeast strains secreting antibacterial compounds.

## EXPERIMENTAL

**The yeast and yeast-like fungi collection**

The collection of yeast, containing over 3,000 samples, was gathered during several expeditions led by V.P. Stepanova and B.F. Yarovoy to extreme regions of Russia: the Kuril Islands, the Kamchatka Peninsula in August–September 1988, 1989, and 1994, and the Sakhalin Island in August–September 2004 [15]. The microorganisms in the collection were obtained from substrates, such as living plants, fallen parts of plants, soil, and insects. Substrates were collected on the sides of volcanoes, near the active zones, and in river and creek valleys.

The yeast isolates were obtained under laboratory conditions. Long-term storage was done at –80°C in the Yeast Peptone Dextrose (YPD) Broth (Sigma- Aldrich, USA) supplemented with 25% glycerol [16].

At the initial stage of collection description, 98 randomly selected strains were identified using the morphological and biochemical approaches [17, 18]. Among the selected strains were representatives of 20 known species: *Candida haemulorni, Candida sake, Candida sorbosivorans, Cryptococcus albidus, Cryptococcus hungaricus, Cryptococcus laurentii, Debaryomyces hansenei, Metschnikowia reukaufii, Pichia farinosa, Rhodotorulla aurantiaca, Rhodotorula glutinis, Rhodotorula minuta, Rhodotorula mucilaginosa, Saccharomyces cerevisiae, Sporobolomyces roseus, Sporidiobolus salmonicolor, Torulaspora delbrueckii,* and *Tremella foliacea*, as well as one member of each of the genera *Bullera *and *Trichosporon*. These species belong to all three known classes of fungi: (1) ascomycetes – 6 species; (2) basidiomycetes – 2 species; and (3) deuteromycetes – 12 species. The species diversity of the collection closely tracks the data on the characteristic yeast species composition of the northern latitudes of Western Siberia and Alaska [19].

Several samples of the collection have already been shown to be able to absorb various types of pollutants, such as radionuclides and heavy metal ions [15, 20].

**Preparation of yeast culture liquids**

The yeast strains were grown on the surface of a YPD agar plate for 3 days at room temperature. The culture was observed morphology-wise to ensure cellular purity. The cells were transferred into a YPD liquid medium and incubated at room temperature for 3 days on a shaker. Culture liquids were separated from the cells by centrifugation at 4 000 g for 15 min at 4°C using a Union 5KR centrifuge (Hanil Science Industrial) and concentrated 15 to 20-fold using a Concentrator Plus centrifuge concentrator (Eppendorf AG) at room temperature for 8–9 h. The concentrated culture liquids were stored at –20°C.

**Double fluorescent protein reporter for identification of the substances causing bacterial ribosome stalling or DNA damage**

The double fluorescent protein reporter plasmid pDualrep2 carries the *rfp *gene under the control of the SOS-induced *sulA *gene promoter and the gene of the Katushka2S protein downstream of the modified tryptophan attenuator (tryptophan codons are replaced with alanine ones) under the control of the constitutive T5 promoter [9, 21]. Modification of the tryptophan attenuator results in a disruption in the movement of the ribosome that is due to specific translation inhibitors rather than to tryptophan starvation.

**Table 1:** The primers used for taxonomic identification

Regions	Primer sequences [22]
ITS1-5.8S-ITS2	ITS1 (forward) 5’- TCCGTAGGTGAACCTGCGG-3’
	ITS4 (reverse) 5’-TCCTCCGCTTATTGATATGC-3’
26S rDNA (part 1)*	LROR (forward) 5’- ACCCGCTGAACTTAAGC-3’
	LR3 (reverse) 5’-CCGTGTTTCAAGACGGG-3’
26S rDNA (part 2)*	LR3R (forward) 5’-GTCTTGAAACACGGACC-3’
	LR7 (reverse) 5’-TACTACCACCAAGATCT-3’

^*^The PCR product of 26S rDNA obtained with the primers LROR and LR7 contains more than 1,000 bp and was obtained with four primers.

The *E. coli *JW5503 strain (lacking the *tolC *gene coding for the essential component of the efflux system) transformed by the reporter plasmid pDualrep2 (AmpR) synthesizes RFP in the presence of DNAdamaging (SOS response-causing) agents; and Katushka2S, in the presence of translation-stalling chemicals [9]. The fluorescent signal of RFP becomes detectable at 574 nm upon excitation at 553 nm; for Katushka2S, at 633 and 588 nm, respectively.

**Analysis of antibacterial activity using the reporter system**

The reporter bacterial culture was grown until OD_600_ = 0.5–1 at 37°C in a LB medium containing 100 μg/mL ampicillin and stored at 4°C overnight. The next day, the culture was diluted with a fresh LB medium to OD_600_ = 0.1. Wells of a 96-well culture plate were filled with 200 μL of the cell culture. The sample was added to the bacteria suspension in the wells. The culture plate was incubated in a plate shaker-thermostat at 37°C. The various degrees of fluorescence for reporter proteins RFP (553/574 nm), Katushka2S (588/633 nm), and OD_600_ in the culture plate were measured in an EnSpire 2300 plate reader (Perkin Elmer). The results were analyzed using the GraphPad Prism 6.0 software.

To validate our system, 2 μL of known antibiotics at sublethal concentrations were added into the wells with the bacteria suspension. We chose several translation inhibitors with different modes of action, such as gentamicin, chloramphenicol, fusidic acid, neomycin, hygromycin B, kanamycin, puromycin, tetracycline, erythromycin, and streptomycin. The panel of drugs inducing the SOS response consisted of nalidixic acid, levofloxacin, ciprofloxacin, and rifampicin.

The concentrated culture liquids (5 μL) were added into the bacterial suspension in the wells of the culture plate. In this dosing manner, sample concentration in the plate’s wells was 2–2.5 times lower than that of the original, non-concentrated sample. The samples with the best inhibitory properties were tested in triplicate.

**Data processing**

We calculated the S/S0 ratio, where S is the reporter fluorescent signal or OD_600_ of the reporter bacterial culture in the presence of a test sample and S0 is the reporter fluorescent signal or OD_600_ of the bacterial culture without additives.

A set of S/S0 values for control was obtained to calculate the background interval. The distribution normality was confirmed with a confidence level of 99% using the D’Agostino–earson omnibus test in the GraphPad Prism 6.0 software. The background interval was defined as [mean-3σ; mean+3σ]. Background intervals: SOS activation signal after 5 h, [0.87; 1.13]; SOS activation signal after 24 h, [0.82; 1.18]; translation inhibition signal after 5 h, [0.93; 1.07]; translation inhibition signal after 24 h, [0.85; 1.15]; and OD_600_ after 5 h, [0.95; 1.05]; OD_600_ after 24 h, [0.97; 1.03].

**Taxonomic identification**

For the taxonomic identification of the yeast isolates, cell walls were destroyed by zymolyase (Zymo Research) in 1 M sorbitol and 0.1 M EDTA (pH 8.0). Total genomic DNA was extracted using a DNeasy Blood & Tissue Kit (Qiagen) and visualized by 1% agarose gel electrophoresis. The ITS1-5.8S-ITS2 and D1/D2 domains of the 26S rDNA (nrLSU) [22] were amplified using the primers listed in *Table 1*. The PCR products were purified using the NucleoSpin Gel and a PCR cleanup kit (Macherey-Nagel). The same primers were used for sequencing. The sequencing results were processed using The Basic Local Alignment Search Tool (BLAST) (http://www.ncbi.nlm.nih.gov/ BLAST).

## RESULTS

**Validation of the reporter system using a set of known antibiotics**

We chose a number of inhibitors with different mechanisms of action as we tested the specificity of the analysis of antibacterial activity using the reporter system in a liquid medium. This choice ensured that all the antibiotics could suppress bacterial growth, whereas the synthesis of reporter proteins happened selectively as a result of ribosome stalling (expression of the fluorescent reporter protein Katushka2S) or events that damaged the DNA (expression of RFP). We used five to six sublethal concentrations of the antibiotics to visualize the profile of the reporters’ expression.

**Fig. 1:**
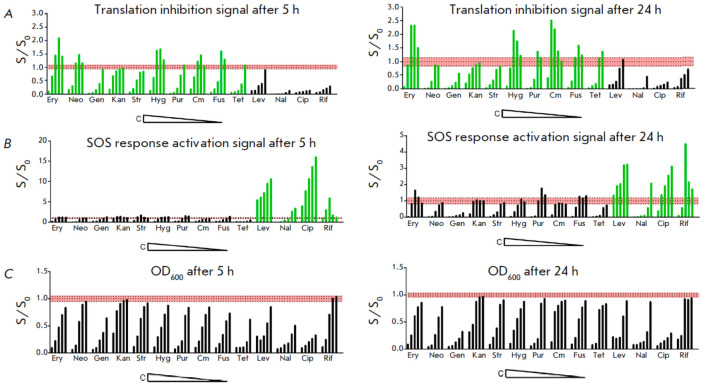
Testing the double fluorescent protein reporter assay on known antibiotics. The vertical axis shows the S/S0 ratio, where S is the reporter fluorescent signal or OD_600_ detected in the presence of a sample; S0, without the sample. The horizontal axis shows the applied antibiotics; for each antibiotic, the concentrations (C) are arranged in descending order. Erythromycin (Ery) 10, 5, 2.5, 1.25, and 0.6 μg/mL; neomycin (Neo) 20, 10, 5, 2.5, and 1.25 μg/mL; gentamicin (Gen) 20, 10, 5, 2.5, and 1.25 μg/mL; kanamycin (Kan) 30, 15, 7.5, 3.8, and 1.9 μg/mL; streptomycin (Str) 12.5, 6.3, 3.1, 1.6, and 0.8 μg/mL; hygromycin B (Hyg) 100, 50, 25, 12.5, and 6.3 μg/L; puromycin (Pur) 15, 7.5, 3.8, 1.9, and 0.9 μg/mL; chloramphenicol (Cm) 0.7, 0.35, 0.18, 0.09, and 0.04 μg/mL; fusidic acid (Fus) 10, 5, 2.5, 1.25, and 0.6 μg/mL; tetracycline (Tet) 1.2, 0.6, 0.3, 0.15, and 0.075 μg/mL; levofloxacin (Lev) 0.1, 0.05, 0.025, 0.013, and 0.006 μg/mL; nalidixic acid (Nal) 100, 50, 25, 12.5, 6.3, and 3.1 μg/mL; ciprofloxacin (Cip) 1, 0.5, 0.25, 0.13, and 0.06 μg/mL; rifampicin (Rif) 25, 12.5, 6.3, 3.1, and 1.6 μg/mL. The red area indicates the range of background values. (*A*) Ribosome stalling reporter signal after incubation for 5 h (left) and 24 h (right). Green bars mark the ribosome-targeting antibiotics; black ones denote the antibiotics activating the SOS response. (*B*) The SOS response activation reporter signal after 5 h (left) and 24 h (right) of incubation. Green bars mark the antibiotics that activate the SOS response; black ones denote ribosome-targeting antibiotics. (*C*) Cell growth values after 5 h (left) and 24 h (right) of incubation

The vast majority of ribosome-targeting inhibitors can block the particular translation reactions responsible for ribosome arrest. Thus, chloramphenicol (fenicols) and erythromycin (macrolides) bind at the entrance of the nascent peptide exit channel and interfere with peptide bond formation, depending on the length and sequence of a growing amino acid chain [23, 24]. Both antibiotics vigorously activate the expression of Katushka2S (*Fig. 1*). A comparable level of induction of Katushka2S was observed upon the addition of translocation inhibitors: hygromycin B (aminoglycosides) blocks the movement of tRNA during the elongation cycle [25], whereas fusidic acid (fusidines) inhibits the dissociation of EF-G from the ribosome [26]. Tetracycline (polyketides), which binds the small ribosomal subunit and affects tRNA delivery to the ribosome [27], and puromycin, which integrates the growing polypeptide chain causing its premature termination [28], increased the reporter fluorescence level above its baseline after incubation for 24 h.

Aminoglycosides interfere with translation mainly by reducing ribosome selectivity, which results in the insertion of incorrect amino acids into the nascent peptide chain, rather than ribosome pausing [29]. Neomycin induced some degree of reporter fluorescence after 5 h of incubation, followed by the disappearance of the signal at time point 24 h, whereas other members of the group (gentamicin, kanamycin, and streptomycin) did not stimulate Katushka2S synthesis at all.

Addition of quinolones (nalidixic acid, levofloxacin, ciprofloxacin), which block DNA replication [30], and ansamycin (rifampicin), which stops RNA synthesis in the cell [31], reduces the Katushka2S fluorescence level below the background at almost all the tested concentrations. Meanwhile, RFP expression remained substantially upregulated within the entire period of detection; the signal was amplified up to 15-fold compared to that of the untreated cells after 5-h incubation. Interestingly enough, some translation inhibitors also stimulated the emergence of the RFP signal; however, none of them exceeded the S/S0 ratio = 2 during 24 h of incubation.

Hence, 5 h of incubation is enough to draw a conclusion as to whether the tested substance activates the SOS response, whereas detection of the compounds causing ribosome arrest may require an extension of incubation to 24 h.

**Analysis of the antibacterial activity of yeast culture liquids**

**Fig. 2:**
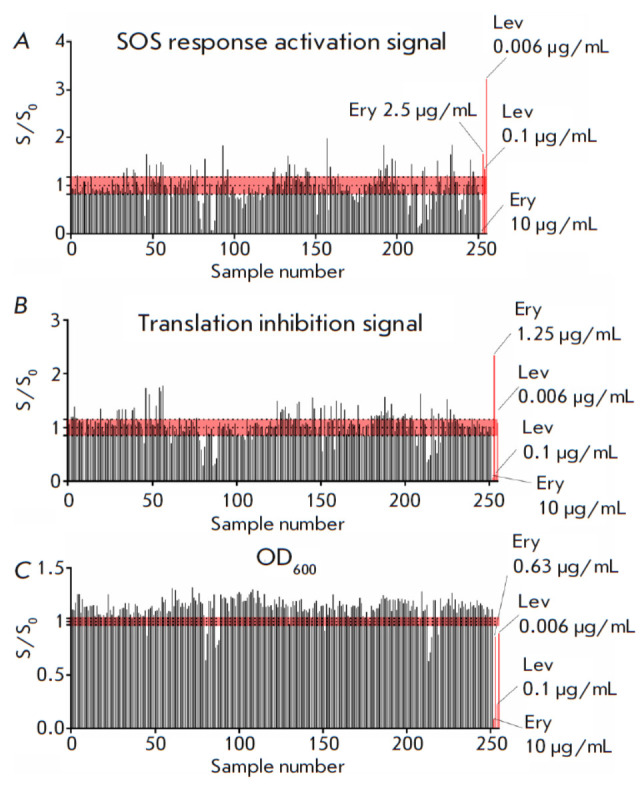
Application of the double fluorescent protein reporter assay on the Sakhalin yeast collection. The vertical axis shows the S/S0 ratio, where S is the reporter fluorescent signal or OD_600_ induced in the presence of a sample; S0 is the reporter fluorescent signal or OD_600_ induced without a sample after 24 h. The horizontal axis shows sample number. Data for erythromycin (Ery) (10, 2.5, 1.25, 0.6 μg/mL) and levofloxacin (Lev) (0.1, 0.006 μg/mL) are used as positive controls. The red area indicates the range of background values. (*A*) The SOS response activation reporter signal. (*B*) Translation inhibition reporter signal. (*C*) The OD_600_ values

We analyzed the antibacterial activity of the entire Sakhalin collection, which contained 251 strains (*Fig. 2*). Eleven samples of culture liquids were deemed to reduce the bacterial growth rate; however, none of them appeared to activate expression of the reporter proteins. Meanwhile, 233 samples were shown to stimulate cell growth, likely due to the presence of some nutritious components. Interestingly enough, among the stimulators we identified 74 samples that appeared to induce ribosome stalling and 50 samples that activate the SOS response. This observation implies a level of complexity of the content of the extracts: relatively low amounts of inhibiting components unable to overcome the probiotic action but still detectable by the reporter system. Hence, moderate antibacterial activity with an unidentified mechanism of action was established for 4% of the Sakhalin collection, while probiotic activity was detected in 93% of the extracts. However, it is possible that fractionation of crude extracts could lead to the detection of additional substances exhibiting antibacterial activity.

**Fig. 3:**
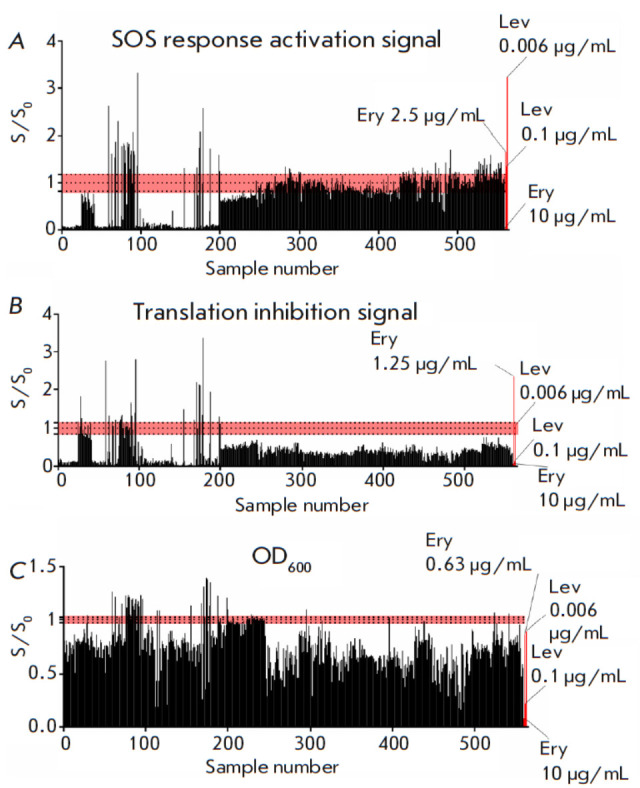
Application of the double fluorescent protein reporter assay on the yeast collection from the Kamchatka Peninsula. The vertical axis shows the S/S0 ratio, where S is the reporter fluorescent signal or OD_600_ induced in the presence of a sample; S0 is the reporter fluorescent signal or OD_600_ induced without a sample after 24 h. The horizontal axis shows the sample number. The data for erythromycin (Ery) (10, 2.5, 1.25, 0.6 μg/mL) and levofloxacin (Lev) (0.1, 0.006 μg/mL) are used as positive controls. The red area indicates the range of background values. (*A*) The SOS response activation reporter signal. (*B*) Translation inhibition reporter signal. (*C*) The OD_600_ values

The yeast collection from Kamchatka contains more than 2,500 samples. We tested 559 of them, and 482 appeared to suppress cell growth, while 41 stimulated it, and 36 had no impact (*Fig. 3*).

We uncovered two samples that induce expression of Katushka2S, 46 samples that stimulate RFP expression, and one sample that activates both reporter signals simultaneously with cell growth suppression. Both Katushka2S inducers demonstrated a decreased RFP signal, and all 46 samples that increase the SOS response signal appeared to lower the translation inhibition reporter signal. It is reasonable to assume that these extracts may contain ribosome stalling and DNA damaging substances, respectively. The sample that stimulates the expression of both reporter proteins has a profile that resembles that of ribosome stalling antibiotics rather than of SOS response inducers.

In the group of cell growth inhibitors, 471 samples appear to push the Katushka2S signal below its baseline. For about half of them (213 extracts), the expression of RFP stood at the baseline level. Similar effects were produced by translation inhibitors that do not lead to ribosome stalling (*Fig. 1*). Eight samples were shown to inhibit cell growth without affecting the Katushka2S signal, reducing the RFP signal to a level comparable to that of given concentrations of neomycin and tetracycline. This case may be indicative of the presence of an inhibitor that does not induce a SOS response but whose mechanism of action could be associated with translational impairment.

Among the extracts that enhance cell growth, we found 21 samples that induce ribosome stalling and 30 that activate the SOS response. We also identified one sample that activates the SOS response but did not impair cell growth. Potentially, these extracts contain low concentrations of antibacterial components.

Hence, contrary to the Sakhalin collection, only 7% of the tested collection from Kamchatka was shown to exhibit probiotic activity. Antibacterial action of varying intensity was observed for 86% of the evaluated samples; a number of extracts caused the activation of the SOS response and ribosome stalling.

Most of the inhibiting extracts had comparable fluorescence signal profiles, with significantly decreased Katushka2S expression and a near-baseline RFP level. Similar behavior was demonstrated by the translation inhibitors that did not cause ribosome stalling, suggesting that the behavior may be a potential inhibition mechanism exploited by yeast extracts.

Among all the studied samples, 44 from Kamchatka exhibited pronounced antibacterial properties (OD_600_ of the bacterial culture was more than twofold lower compared to the reference). The substrate collection spots in Kamchatka were chosen in close proximity to mud, water, or gas emissions: the Pauzhetka, Ozernovsky, Mutnovsky, Esso settlements and their environs, the vicinity of Skalistaya Hill and Kuril Lake. Twenty-eight of the 44 isolates were found in the gorge located about 200 m away from the Pauzhetka geothermal power plant; 11 isolates, on thistle (*Onopordum acanthium*); and 17 isolates, on wormwood (*Artemisia vulgaris*).

Knotweed, bluegrass, columbine, speedwell, etc. were also collected in this gorge; however, their yeast isolates did not exhibit any pronounced antibacterial properties. Wormwood and thistle were also collected in other areas: wormwood, in Tikhaya Bay of Kuril Lake (one isolate with pronounced antibacterial properties), on the coast of the Sea of Okhotsk in the vicinity of the Ozernovsky settlement, on flatland near the Pauzhetka settlement, on the slopes of Skalistaya Hill (no isolates with pronounced antibacterial properties); thistle, in Tikhaya Bay of Kuril Lake, on the slopes of Skalistaya Hill (no isolates with pronounced antibacterial properties), on the flatland near the Mutnovsky settlement (one isolate with pronounced antibacterial properties).

The culture liquids of 43 out of the 44 isolates exhibiting pronounced antibacterial properties reduced reporter signals below their control level; i.e., they did not induce activation of the SOS response or ribosome arrest.

**Taxonomic identification**

We selected 19 samples of cultural liquids that suppressed cell growth with the S/S0 ratio lying in the range of 0.17–0.97. Three samples (with the collection numbers KI-55-1-9-1, KI-55-1-9-3*, and KI-53-1-13a*) appeared to possess the strongest inhibitory properties; their S/S0 values lay in the range of 0.17–0.29. Four samples (with collection numbers KI-1-1, KI-3-6a, KI-19-1a, and KI-31-3) provoked an increase in the SOS response activation reporter signal.

This group was shown to contain predominantly members of the *Cryptococcus (Naganishia) *genus (*Table 2*). Species diversity was as follows: five strains,* Cryptococcus adeliensis*; four strains, *Naganishia (Cryptococcus) albidosimilis*; two strains, *Naganishia (Cryptococcus) diffluens*; one strain, *Naganishia (Cryptococcus) liquefaciens*; four strains, *Naganishia (Cryptococcus) vishniacii*; two strains, *Candida parapsilosis*; and one strain, *Rhodotorula mucilaginosa*.

The yeast isolates KI-55-1-9-1, KI-55-1-9-3*, and KI-53-1-13a*, which exhibit the most vigorous antibacterial activity, belong to the *Naganishia (Cryptococcus) albidosimilis *and *Naganishia (Cryptococcus) adeliensis *species*. *The strains KI-1-1, KI-3-6a, KI-19-1a, and KI-31-3, whose culture liquids stimulated the SOS response, belong to the* Naganishia (Cryptococcus) albidosimilis, Naganishia (Cryptococcus) adeliensis*, and *Candida parapsilosis* species*.*

**Table 2:** The results of the taxonomic identification of yeast strains whose culture liquids exhibited antibacterial properties

Collection number of a strain	Species affiliation of a strain / the corresponding BLAST sequence number
KI-1-1	Cryptococcus adeliensis / JX188117.1
KI-3-6a	Cryptococcus adeliensis / JX188117.1
KI-19-1a	Candida parapsilosis / KT282393.1
KI-174-4a	Candida parapsilosis / KT282393.1
KI-17-5-1a	Rhodotorula mucilaginosa / MN006694.1
KI-18-1a	Naganishia diffluens / MK793259.1, MT303133.1
KI-53-1-6d	Cryptococcus adeliensis / JX188117.1
KI-80-1	Naganishia liquefaciens / MG722803.1
KI-81-2-1	Naganishia vishniacii / OM337523.1
KI-55-1-1**	Naganishia diffluens / MK793259.1, MT303133.1
KI-46-5c-2	Naganishia albidosimilis / MW248429.1, MT127371.1
KI-31-3	Naganishia albidosimilis / MW248429.1, MT127371.1
KI-39-5	Naganishia vishniaci / OM337523.1
KI-151-0	Naganishia vishniacii / OM337523.1
KI-223-1b	Naganishia vishniacii / OM337523.1
KI-193-3	Cryptococcus adeliensis / JX188117.1, JX188114.1
KI-55-1-9-1	Naganishia albidosimilis / LC203701.1, LC203699.1, MW248429.1
KI-55-1-9-3*	Naganishia albidosimilis / LC203701.1, LC203699.1, MW248429.1
KI-53-1-13a*	Naganishia adeliensis / JX188117.1, JX188114.1, LC202041.1

## DISCUSSION

The current extinction rates of our biodiversity are estimated to be approximately 100 to 1,000 times higher than those over the past centuries [32]. The ongoing loss of biodiversity results in the disappearance of at least one important bioactive molecule every two years [33]. The discovery and storage of biomaterial, with subsequent organization of species collections, followed by investigations of these collections, contribute to efforts at biodiversity preservation [2, 3].

In this study, we have analyzed the antibacterial activity of yeast strains found in the Kamchatka Peninsula and Sakhalin Island against reporter *E. coli* Δ*tolC *cells. The absence of the *tolC *gene improved assay sensitivity [9, 34] by increasing compound dissemination into the cell, which is highly preferable for multicomponent biological crude extracts with potentially low concentrations of bioactive compounds.

High-throughput screening of bioactive substances is usually performed on bacterial lawn using agar plates [35]. However, bacterial cultures on solid media and in liquid differ metabolically. The proteomes of a single *E. coli *colony have only 68% protein overlap in the case of two culturing conditions [36]. Hence, despite the widespread use of the dual-fluorescent reporter system on solid media [9], it was important to validate its application in a liquid medium using a set of known antibiotics. Notably, SOS response activators dramatically increased the RFP fluorescence after 5 h of incubation with bacterial cells, whereas differentiation of translation inhibitors into ribosome stalling and miscoding agents was possible only after 24 h into the experiment. In general, our results are in line with the previously obtained findings in [9], proving the applicability of this system in liquids.

The modification to the assay introduced offers several advantages. First, it allows one to monitor the density of the cell culture by measuring OD_600_, enabling the evaluation of the probiotic and antibacterial properties of a sample. It also makes it possible to add a test substance at different bacterial growth phases. This might be relevant as bacterial sensitivity to antibiotics depends on the metabolic state of the cell [37]. Second, it is possible to record both an increase and a decrease in the reporter signal with respect to the reference level. Reduction of the fluorescence level, accompanied by lowering of OD_600_, may indicate cell death, whereas a decrease in the fluorescence followed by an increase in OD_600_ over the starting values may indicate profound changes in the cellular metabolism [24]. Third, the “drug–bug” race begins on an agar plate when a testing sample meets the medium [35]. Sample molecules diffuse into agar, creating a dynamically changing concentration gradient, while bacterial growth progresses along the gradient. The result is a competition of cell growth rates and diffusion rates of the test drug. In the case of samples with low bioactive concentrations, this effect may impair the analysis. Meanwhile, the assay for the analysis of antibacterial activity in a liquid medium has several drawbacks such as a longer processing time, greater amount of consumables, and the impossibility of analyzing poorly soluble substances.

We analyzed the antibacterial properties of the yeast collection and compared the data obtained for samples from Sakhalin and Kamchatka. Interestingly, no significant antimicrobial properties were found in the Sakhalin samples; on the contrary, the samples were predominantly probiotics. Strains from the Kamchatka Peninsula exhibited strong antibacterial properties, and several strains led to the activation of reporter signals in the test system. However, 43 of the 44 isolates with the strongest antibacterial properties did not lead to the activation of the SOS response and ribosome stalling, suggesting that the bioactive substances released may disrupt the bacterial membrane integrity or suppress the ability of bacteria to form biofilms [38]. In the present study, yeast culture liquids were obtained without the destruction of yeast cells; therefore, it is most likely that wild yeasts would excrete biologically active metabolites into the extracellular space.

Taxonomic identification of yeast strains revealed that isolates exhibiting the strongest antimicrobial properties belong to the *Naganishia (Cryptococcus)* genus*. *This agrees with previous reports on the toxicity of yeast isolates of the *Naganishia (Cryptococcus)* genus discovered in various geographical areas. The* Naganishia albida *and *Naganishia diffluens *species found in the alkaline water lake region Wadi El- Natrun, Egypt, were found to possess antibacterial activity against *E. coli *and *Staphylococcus capitis *[39]; a probable pathogenicity of the *Naganishia adeliensis* strains for living organisms and their production of secondary metabolites, including mycotoxins, was reported for yeast discovered in the vicinity of Lodz, Poland [40].

We established that more than half of the strains with pronounced antibacterial properties were collected in the gorge near the Pauzhetka geothermal power plant on thistle and wormwood. Moreover, it was the combination of the plants and their growth sites that led to the emergence of this bioactivity in yeast culture liquids. The gorge near Pauzhetka village is a biotope combining high humidity, low light, and soil heating from hydrothermal vents. Such conditions foster a significant biodiversity of microorganisms. Yeasts are known to be able to protect the host plant against mycopathogens by attaching to the surface of fungi and secreting enzymes that destroy the mycopathogens’ cell walls [38]. In the case of high competition, the concentration of these yeast enzymes can be increased and have a destructive impact on bacterial membranes as well. Meanwhile, wormwood and thistle contain bioactive sesquiterpene lactones [41, 42], which possesses antimycotic properties [43]. Comfortable environmental conditions could induce yeast cells to develop resistance to these compounds, for example, by synthesizing enzymes capable of modifying sesquiterpene lactones to a safe form, or by synthesizing low-molecular-weight components that disrupt the biosynthesis of sesquiterpene lactones. These bioactive molecules could also have a negative effect on bacterial cells. It is known that plants with antimicrobial properties “cultivate” mutualistic microflora with similar bioactivity. Thus, the antibacterial potential of pomegranate peel can be determined not only by the nature of its own components, but also by microorganisms, and local yeast, in particular [44].

The true mechanisms by which yeast culture liquids apply antibacterial action can be determined only after the isolation and identification of bioactive components. But the uniqueness of the discovered biocenosis in this study is beyond doubt and can be used as a template for similar expeditions. The researchers note the need to analyze interactions not only within individual groups of microorganisms (bacteria with bacteria or yeasts with yeasts), but also the interactions between groups for a deeper understanding of how they function and the environmental impact they have [45]. Our work contributes to the research into the interactions that exist in microbial communities within certain biocenoses, emphasizing the importance of using an integrated approach when searching for biologically active substances.

## CONCLUSIONS

We were able to identify the strains of a yeast collection that exhibit antibacterial and probiotic properties. That discovery affirms the value of the uniqueness of the Far East collection and demonstrates the need to preserve and research biodiversity.
